# Entropy Production Analysis and Fluid–Structure Refinement of a Stepless Stratified Intake

**DOI:** 10.3390/e28030256

**Published:** 2026-02-26

**Authors:** Jiahuan Qi, Ke Liu, Xingen Wang, Jianping Zhao, Jun Li

**Affiliations:** 1 Nanjing Hydraulic Research Institute, Nanjing 210029, China; jiahuan_qi@hhu.edu.cn (J.Q.); jpzhao@nhri.cn (J.Z.); 2 Science and Technology Research Centre, China Yangtze Power Co., Ltd., Wuhan 430010, China; 3 PowerChina Guiyang Engineering Corporation Limited, Guiyang 553304, China; wangxe_gyy@powerchina.cn

**Keywords:** stepless stratified intake, selective withdrawal, entropy production, hydraulic losses, refinement, flow–structure interaction

## Abstract

Thermal stratification in deep reservoirs can cause ecologically problematic cold-water releases, and many existing selective-withdrawal phenomena rely on a limited set of fixed intake levels, which constrains their ability to follow seasonal shifts in the thermocline. Stepless stratified intakes with continuously adjustable flap gates offer quasi-continuous control of withdrawal depth, but their multi-gate, multi-brace layouts generate complex internal hydraulics whose energy-loss mechanisms are not well captured by conventional head-loss and resistance-coefficient metrics. In this study, physical-model measurements are combined with a validated three-dimensional numerical model, and entropy-production theory is used as a diagnostic to resolve where and by which mechanisms mechanical energy is irreversibly degraded inside a single-unit stepless stratified intake. The analysis shows that turbulent entropy production accounts for more than 98% of total dissipation, concentrated mainly in the flow channel and gate shaft, while the reservoir and outlet pipe contribute only weakly. Local entropy-production-rate fields indicate that dominant irreversibilities are associated with flow turning at the active gate leaves and with separation and wake development around horizontal and vertical braces, which generate low-velocity bands across gate levels and a low-velocity corridor in the shaft. Five geometric modification schemes targeting gate-entrance shaping and brace layout are evaluated; a combined brace-alignment and edge-rounding configuration most effectively weakens dissipation hotspots, improves discharge sharing among gate levels and reduces total entropy production. These findings show that entropy-based diagnostics can complement traditional hydraulic indicators and provide effective guidance for the design and refinement of stepless stratified intake structures.

## 1. Introduction

Thermal stratification develops seasonally in deep reservoirs, strongly affecting downstream ecological conditions. The release of cold hypolimnetic water can depress river temperatures over long distances, altering fish metabolism, shifting spawning and migration cues, and reducing habitat suitability during critical life stages [1,2,3]. Climate-induced warming has been shown to intensify and prolong stratification, making it increasingly difficult to maintain suitable discharge temperatures under hydropower operation [4,5]. In response, selective withdrawal has become an important operational strategy. It allows operators to target specific density layers and actively manage downstream thermal regimes while still generating power and controlling floods [6,7,8,9].

At the engineering level, selective withdrawal is often carried out using various types of intake structures, such as multi-level intakes, stop-log gates, front-wall openings, and thermal control curtains [10,11,12]. These systems access different water layers through multiple discrete openings or internal weirs located at fixed elevations. Previous research has demonstrated that suitable combinations of intake levels and structure types can elevate release temperatures, improve thermal stability, and mitigate eutrophication risks. However, several limitations remain. The vertical spacing between available intake levels is often too large to follow modest shifts in the thermocline, and frequent gate switching complicates scheduling and increases mechanical wear. In deep-water areas upstream of dams, multi-level towers or dense retaining structures can also become bulky and intrusive, leading to high construction and maintenance expenses. As reservoir operation moves toward finer ecological regulation and more flexible multi-objective operation, there is a growing demand for intake systems capable of providing quasi-continuous control of withdrawal depth while maintaining adequate hydraulic efficiency [13,14].

Stepless multi-layer selective-withdrawal devices have emerged in this context as a promising alternative to conventional multi-level structures. However, as an emerging technology, research on its internal hydraulic performance is still in its infancy, with a significant void remaining in the understanding of localized energy dissipation mechanisms. By employing several independently adjustable flap gates that can rotate over a prescribed angle range, such systems allow the withdrawal layer to be shifted in an almost continuous manner within the stratified water column. This offers the potential to track target temperature profiles or ecological flow requirements more closely than is possible with fixed intake elevations. At the same time, the introduction of multiple gate leaves, internal braces, and transition sections greatly increases the geometric complexity of the intake. The coupled flow through the reservoir approach zone, entrance passage, vertical shaft, and downstream conveyance pipe is characterized by complex three-dimensional flow patterns, strong interactions between neighboring components, and pronounced local vortices around structural members. If these internal hydraulics are not adequately understood and controlled, the ecological benefits of stepless selective withdrawal may be offset by unfavorable flow patterns, excessive energy dissipation, and an increased likelihood of hydraulic instabilities.

Existing studies have established a systematic understanding of how thermal stratification in deep reservoirs responds to different withdrawal modes and to climate change. Coupled hydrodynamic–thermal simulations show that alternative intake configurations can trigger markedly different thermal adjustment mechanisms: multi-layer withdrawal tends to reinforce overall stratification, whereas inner-weir withdrawal is more likely to depress the thermocline and increase downstream water temperature by enhancing vertical mixing in the withdrawal layer [15]. Optimizing the withdrawal elevation has also been shown to reduce the risk of bottom hypoxia under persistent stratification or declining water levels by selectively entraining higher-oxygen intermediate water masses [16,17]. In the context of climate warming, the long-term evolution of reservoir thermal structure has attracted increasing attention. Two-dimensional hydrodynamic simulations reveal a trend toward surface warming and enhanced stratification stability, while temperature prediction models highlight increasing extrapolation biases under warming scenarios [18,19]. Related work further indicates that the ability of deep-water models to reproduce the evolution of thermal structure can be significantly improved by refining lake–reservoir–atmosphere parameterization schemes [20]. The withdrawal mode simultaneously modifies local hydrodynamics, influencing both thermal structure and dissolved-oxygen dynamics [21]. For stop-log gate systems, the temperature regulation capacity has been quantified using an equivalent-elevation method, and Fourier spectrum analysis has been applied to characterize the daily variability in deep-reservoir thermal structure and its sensitivity to intake operation [22,23]. Laboratory and field experiments show that selective withdrawal changes the residence time and mixing intensity of near-surface water, which in turn changes the hydraulic conditions that trigger algal blooms [24,25]. At the operating level, optimization models have been utilized to investigate the trade-offs between ecological goals and hydropower generation, to illustrate the efficacy of variable intake elevation in alleviating winter inverse stratification, and to expose the structural deficiencies of traditional stop-log gates for warm-water withdrawal [26,27]. These studies clarify how selective-intake schemes reshape reservoir temperature fields and downstream thermal responses. In contrast, much less attention has been paid to the internal hydraulic losses of selective-intake systems themselves—how dissipation is organized in space, which regions or components dominate the losses, and how these patterns change with withdrawal elevation, particularly in stepless multi-layer devices.

In conventional hydraulic analysis of intake systems, performance is usually evaluated in terms of global quantities such as total head loss, local resistance coefficients, or efficiency indices [28]. These quantities characterize the overall magnitude of loss but provide limited insight into where dissipation is concentrated or which mechanisms dominate in different parts of the flow. Entropy-production theory, grounded in the second law of thermodynamics, provides a complementary perspective: by relating irreversible mechanical losses to local entropy generation, it allows the magnitude and spatial distribution of energy degradation to be evaluated within a unified, thermodynamically consistent framework and to be implemented in CFD post-processing together with high-Reynolds-number turbulence models and wall functions [29,30,31]. A recent review further summarizes the use of entropy-production theory for energy-loss analysis, hydraulic optimization, cavitation assessment and fault diagnosis in pumps and turbines, indicating that the approach has reached a mature level of application in turbomachinery [32].

On this basis, entropy-production analysis has been applied to hydraulic turbines and pump–turbine units to resolve loss mechanisms that are difficult to access from pressure data alone. For Francis and reversible pump–turbine machines, previous studies have used volume-integrated entropy production to reconstruct global hydraulic losses and to decompose these losses among runner, guide-vane passages and the draft tube [33,34]. Local entropy-production fields have also been employed to identify high-dissipation regions associated with backflow, separation vortices and vortex ropes, and to relate their evolution to hump characteristics, S-shaped curves and hysteresis behavior under off-design operation [35,36]. These applications show that total mechanical entropy production can be consistently decomposed into contributions associated with turbulent dissipation, near-wall shear and direct viscous effects, and that the resulting loss budgets track conventional head-loss estimates while simultaneously revealing where and how irreversibilities are generated inside multi-component turbine systems [37].

Entropy-based diagnostics have likewise been introduced in a variety of pump and hydrokinetic devices. In centrifugal and mixed-flow pumps, local entropy-production methods have been used to evaluate energy losses throughout the entire flow passage and to separate the contributions of turbulent and wall-shear dissipation, showing that regions near the blade leading edge, the junction between pressure and suction surfaces and the volute tongue act as persistent centers of high entropy production [38,39]. Parametric studies on low-head and side-channel pumps indicate that changes in impeller–guide-vane spacing and side-channel wrapping angle can markedly redistribute losses among impeller, guide-vane and side-channel domains, providing quantitative guidance for structural optimization [40,41]. In micro horizontal-axis river ducted turbines and related hydrokinetic rotors, entropy-production analysis has been applied to assess how yaw angle and tip clearance affect the balance between useful power extraction and turbulent dissipation, typically revealing intense losses in blade-tip regions, at duct outlets and in downstream wakes [42]. Complementary indicators such as entropy-dissipation and enthalpy–gradient–magnitude metrics have also been proposed to work alongside entropy production, jointly characterizing vortex-induced losses and non-uniform energy distributions in radial-inflow turbines and pumps [43,44,45].

Collectively, the entropy-based analyses of turbines, pumps and hydrokinetic devices demonstrate that entropy-production methods are well suited to diagnosing how hydraulic losses are distributed among components, relating high-dissipation zones to specific flow structures, and supporting geometry and operating-condition optimization in complex internal flows. However, existing applications are almost entirely confined to compact turbomachinery, and there is very limited experience in applying entropy-based diagnostics to large-scale reservoir intake systems or multi-layer selective-withdrawal structures. In particular, the spatial distribution of energy dissipation and its dependence on withdrawal elevation in stepless stratified intakes have not been examined within an entropy-production framework, and the influence of internal braces and gate-leaf geometry on both local high-dissipation regions and system-scale irreversibilities has yet to be quantified.

Building on the well-established application of entropy-production analysis in fluid machinery, the present study extends this theoretical framework to large-scale hydraulic structures to address the existing research void. It integrates entropy-production analysis with physical-model measurements and validated CFD simulations for a full-scale stepless stratified intake. The work pursues three main objectives: (i) to apply entropy-production analysis to quantify how mechanical dissipation is distributed among the reservoir approach, entrance passage, shaft and pipe to identify the regions that systematically act as major sources of irreversible loss; (ii) to analyze how variations in withdrawal elevation, gate-leaf combinations and brace/gate-leaf geometric perturbations reorganize the loss pattern from local components to the system scale; and (iii) to assess geometric refinement schemes and operating configurations designed to reduce unnecessary dissipation while maintaining conveyance capacity and selective-withdrawal performance. Through these objectives, this study aims to provide a new insight into energy-loss mechanisms in stepless stratified intakes and to furnish quantitative guidance for the geometric refinement and hydraulic operation of stepless selective-withdrawal structures.

## 2. Methods

### 2.1. Physical Model

**Figure 1:**
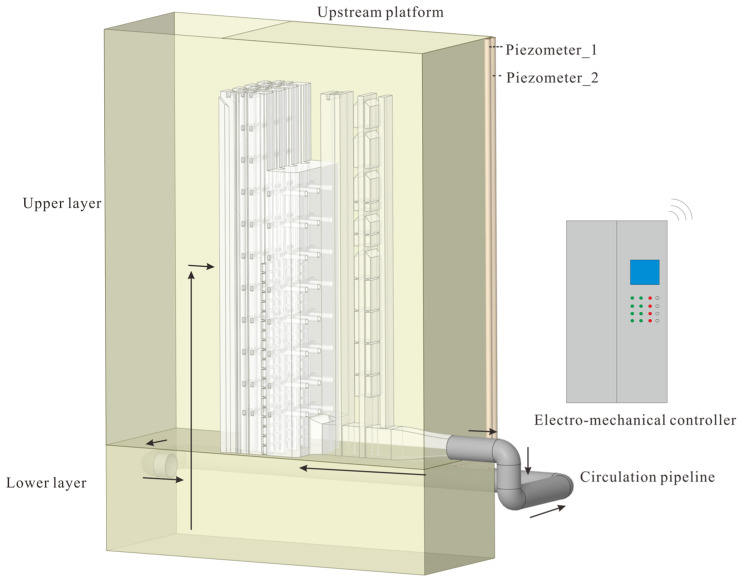
Layout of the physical model of the stepless stratified intake.

**Figure 2:**
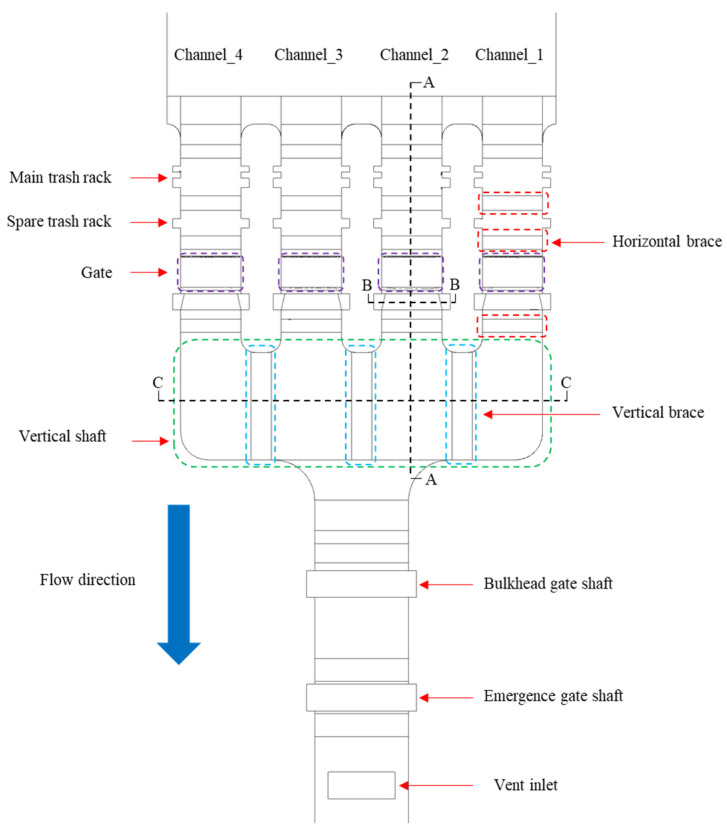
The top view of the main flow channel and gate arrangement.

To replicate the hydraulic behavior of the stepless stratified water intake at a single-unit hydropower station, a 1:20 Froude-scaled physical model was constructed in the Baihetan Hydraulic Laboratory of the Nanjing Hydraulic Research Institute. This scaling ensures geometric similarity and preserves the dominant gravity-driven hydraulic behavior between the prototype and the model. The model includes four main parts: the reservoir, flow channel, gate shaft, and pipe, which represent the complete intake system. Figure 1 shows the overall model layout, Figure 2 presents a top view of the main flow path and structural components, and the principal geometric parameters for both the prototype and the scaled model are summarized in Table 1.

**Figure 3:**
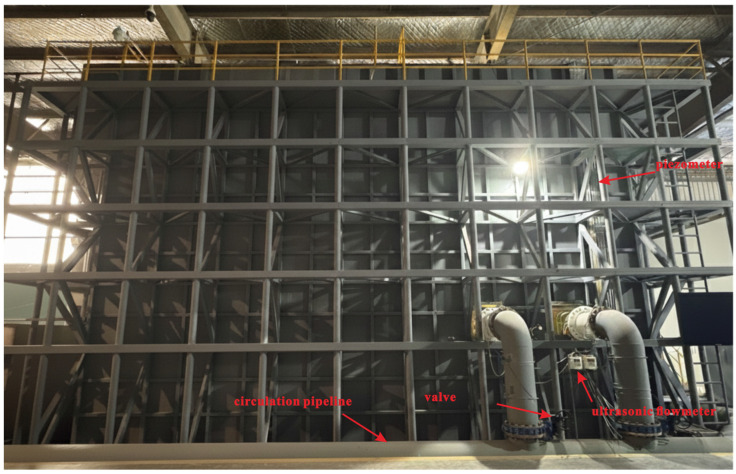
Dual-layer tank with recirculating water-supply system.

**Figure 4:**
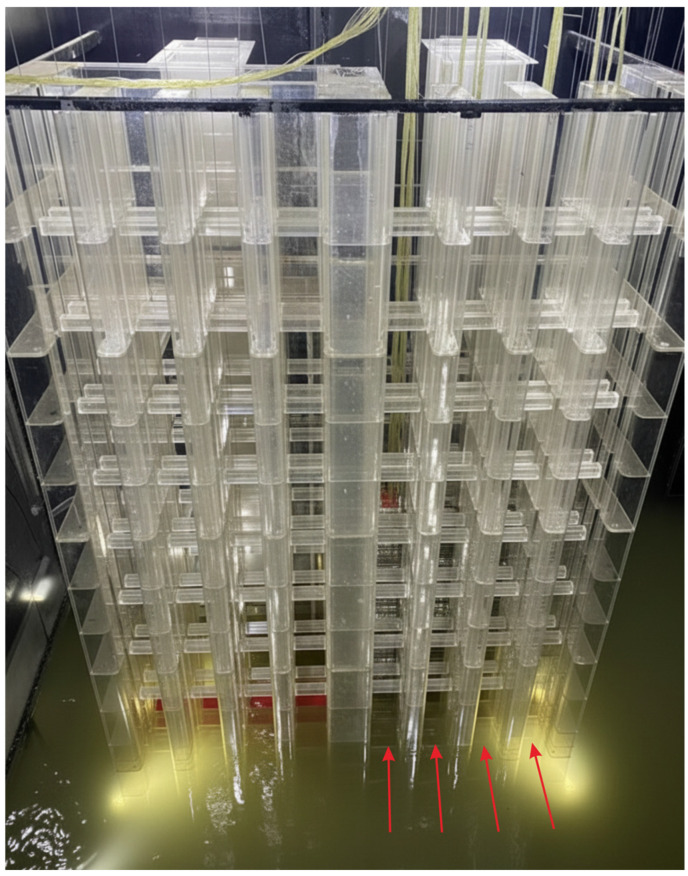
The flow pattern of water in the physical model. (The stepless stratified intake used in this experiment is located on the right side).

**Figure 5:**
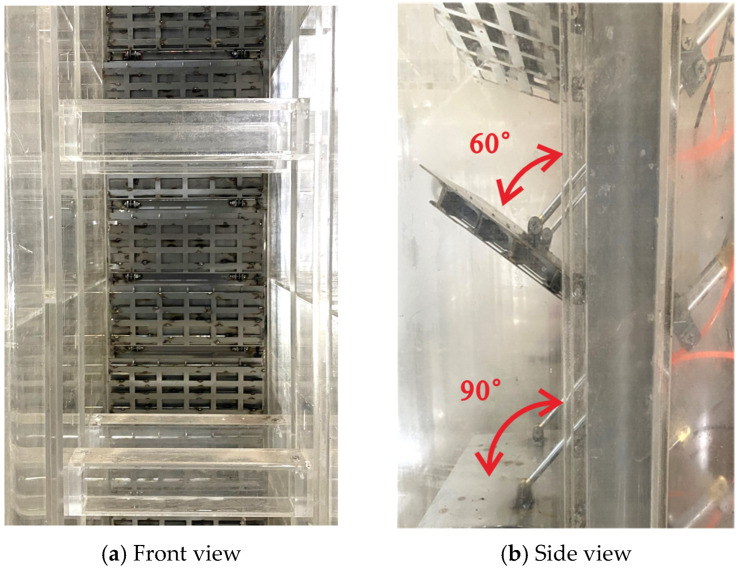
Flap-gate assembly and rotation range in single flow channel.

**Figure 6:**
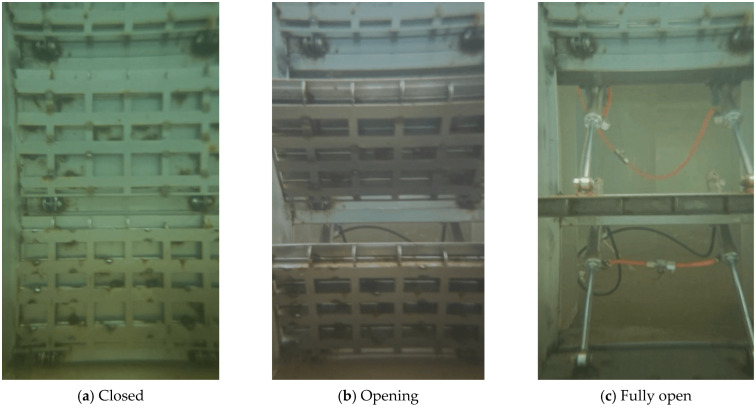
The underwater opening and closing process of the flap gate.

**Figure 7:**
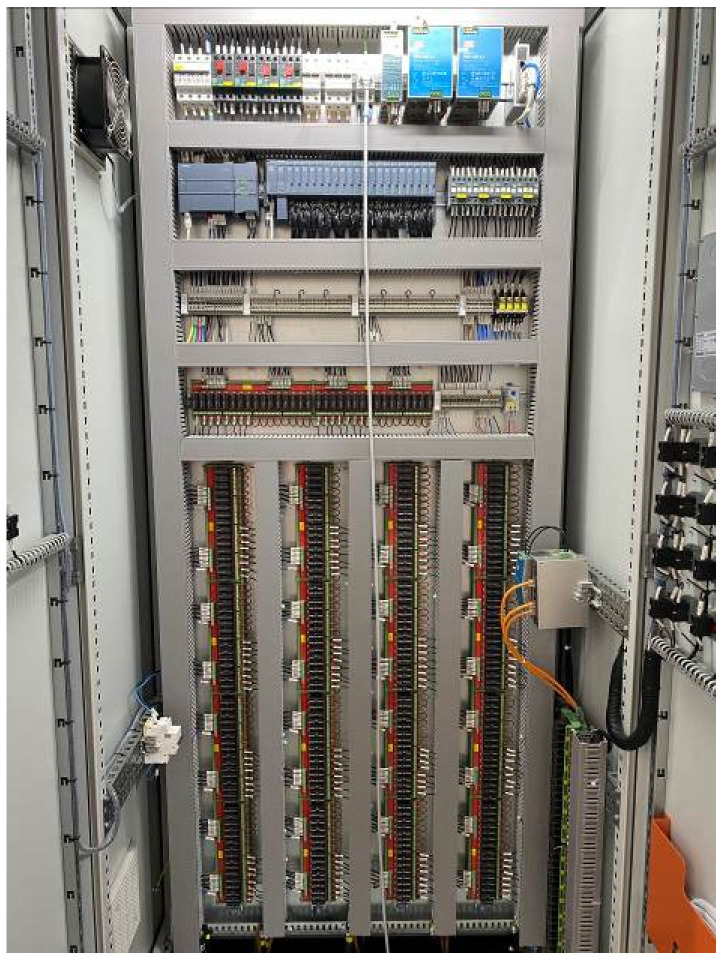
Electro-mechanical controller.

The model is fabricated from transparent acrylic and is installed in a dual-layer experimental tank (Figure 3). The upper layer accommodates the model (Figure 4), while the lower layer serves as a storage basin. Water flows through the intake system, collects in the lower basin, and is pumped back to the upper tank via a circulation pipeline, forming a closed-loop circulation system. The stepless stratified water intake structure is installed in vertical gate slots on both sides of the flow channel. Each channel contains 20 gates arranged in segments along the flow direction. Each gate can rotate from 0° (closed) and 90° (fully open) (Figure 5 and Figure 6), and its openings can be controlled independently or synchronously via electro-mechanical controller (Figure 7), allowing continuous adjustment of the withdrawal depth. Figure 5 illustrates the structural arrangement and rotation mechanism of the flap-gate system, and Figure 6 shows the underwater opening and closing process of the flap gate in single channel.

**Figure 8:**
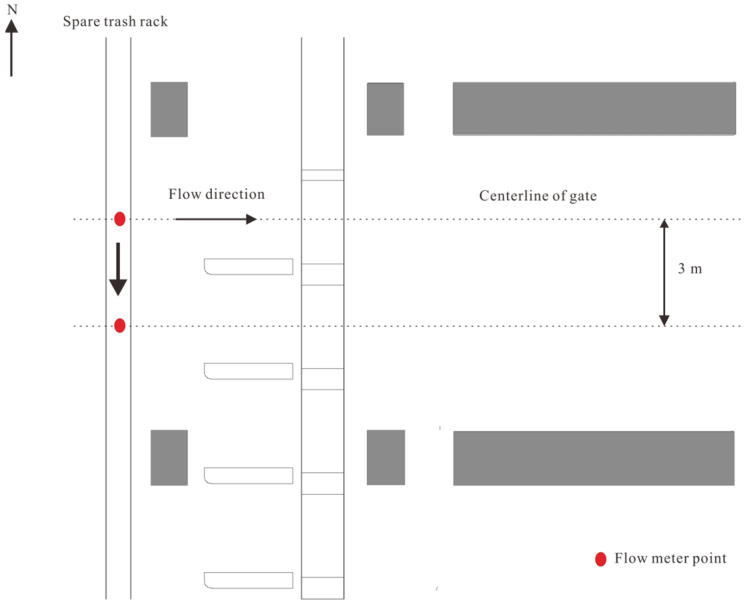
Schematic arrangement of velocity measurements along the flap-gate frame centerline.

The experimental system measures velocity distribution and total head loss under various operating conditions. The measurement setup is shown in Figure 8. Flow velocities are recorded using propeller-type current meters positioned along the centerline of the opened flap-gate frames, accessed through the spare trash rack. During the tests, the current meters are traversed vertically from the top working platform to measure velocities at all designed points. Two piezometric tubes are installed, one in the upstream reservoir and the other in the downstream pipe, to measure the head difference across the intake system. The upstream tube also monitors the reservoir level, which is adjusted by varying the pump frequency and valve opening in response to the measured water level. This procedure maintains a stable hydraulic head at the intake entrance. An ultrasonic flowmeter on the outlet pipe measures discharge, and the flow rate is adjusted by changing the valve opening to match prototype operating conditions.

Under prototype-equivalent hydraulic conditions, four representative operating regimes are established to investigate the stratified-withdrawal behavior of the stepless stratified intake system. The experimental discharge is approximately 165.86 × 10^−3^ m^3^/s, which corresponds to the prototype rated discharge of 296.7 m^3^/s at the 1:20 geometric scale. The submergence depth at the intake is set to 0.9 m (equivalent to 18 m in the prototype) and is kept constant in all tests to maintain consistent hydraulic boundaries at the intake.

**Figure 9:**
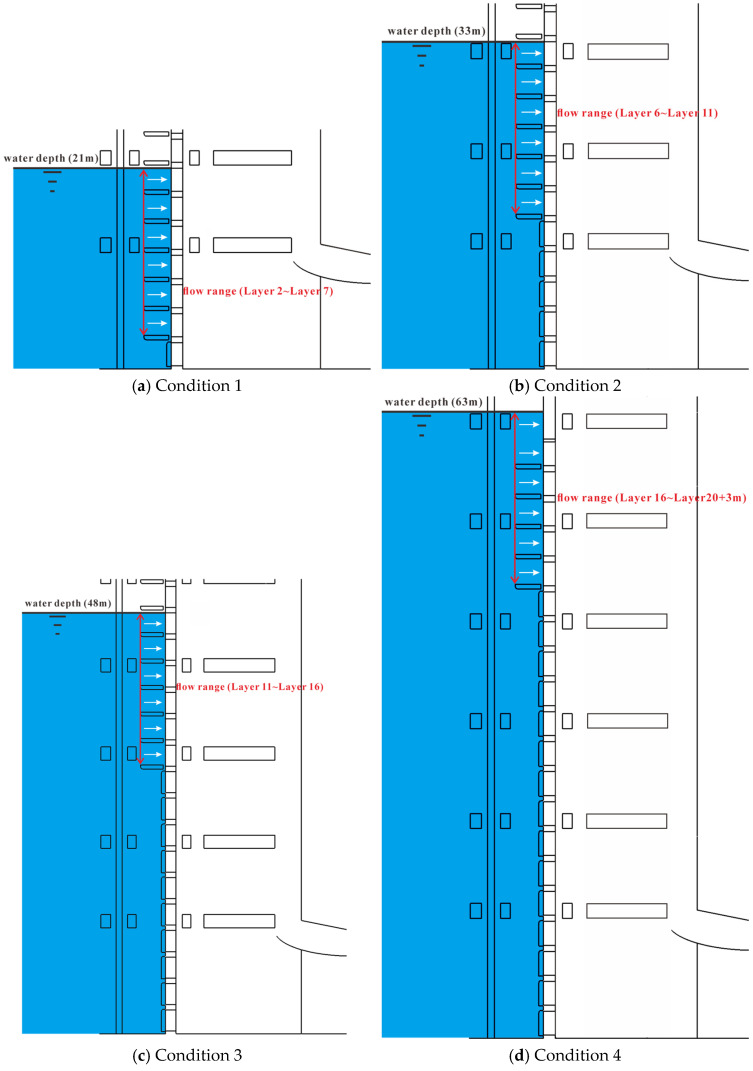
Schematic configurations of the four operating conditions of the stepless stratified intake. White arrows indicate the flow direction; red arrows indicate the flow range.

The model features four parallel flow channels, numbered 1 to 4 from left to right, A-A section is the central side section of the Channel_2, B-B section is the vertical cross section of the gate frame, and C-C section is the central vertical longitudinal section of the vertical brace (Figure 2). Due to the structural symmetry of the intake, observations and measurements focus mainly on the longitudinal sections of Channels 1 and 2 and on the transverse section through the flap-gate frame centerline. The flapped gates in each channel operate synchronously, with each test condition corresponding to a specific combination of gate openings for different stratified-withdrawal modes. In all scenarios, the opened gates are fixed at 90°, while the closed gates remain fully shut. Gate-opening configurations for the four operating conditions are illustrated in Figure 9 and summarized in Table 2.

### 2.2. Numerical Model

**Figure 10:**
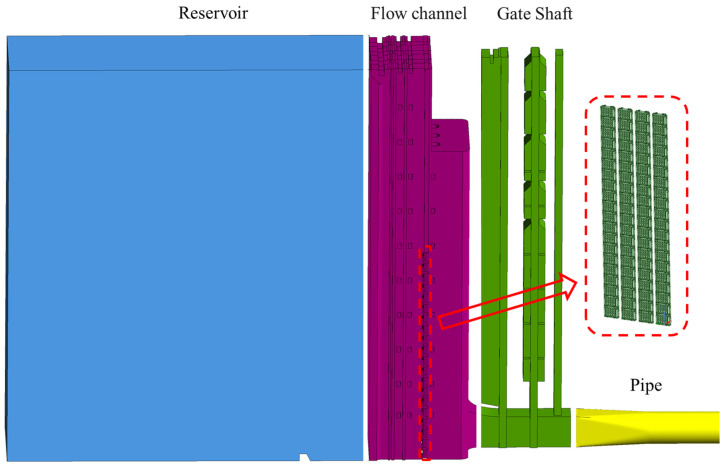
Computational domain of the prototype-scale numerical model.

A three-dimensional numerical model at the prototype scale is developed to simulate the hydraulic performance and internal flow dynamics of a single-unit stepless stratified intake system. The computational domain extends from the upstream reservoir to the downstream pipe, covering the reservoir, flow channel, gate shaft, and pipe (Figure 10). The geometric configuration and operating conditions are kept consistent with those of the physical model, facilitating subsequent validation through comparison with experimental data. The stepless stratified water intake device is modeled as twenty vertically arranged flapped gates mounted within the side slots of the flow channel. The opening states of individual gates are specified according to the operating conditions, in accordance with the experimental configurations listed in Table 2. Minor geometric features, such as fasteners, stiffeners, and small filets, are omitted to enhance computational efficiency, while critical contours, including gate thickness, hinge position, and slots dimensions, are fully retained.

**Figure 11:**
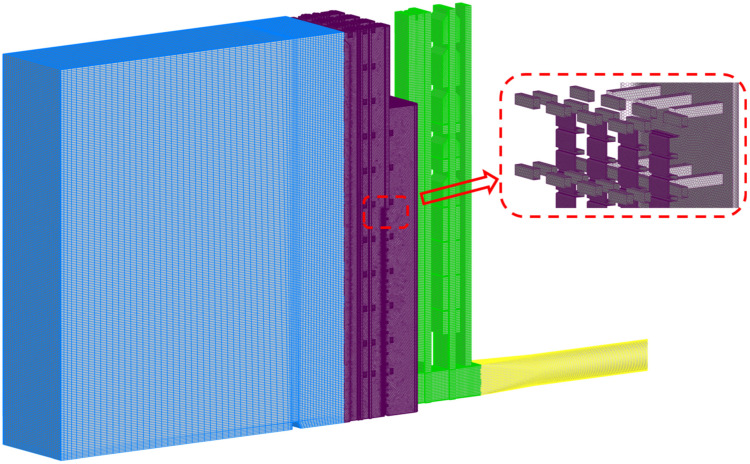
Overall computational mesh of the stepless stratified intake system.

The mesh is generated using ANSYS ICEM CFD 2024 R1 and FLUENT Meshing 2024 R1. Given the geometric complexity of the intake passage, a region-specific meshing strategy is adopted. The reservoir, gate shaft, and pipe are discretized with structured hexahedral grids, whereas the flow channel containing the flap gates and supporting braces is meshed using a hybrid hexahedral-dominant grid. Local grid refinement is applied near the surface of flap gates and horizontal braces, within the minimal cell size kept below 10 mm to better resolve wall shear and recirculation zones (Figure 11). To resolve the near-wall gradients, two inflation layers with a growth rate of 1.2 are implemented, ensuring a smooth transition to the core flow mesh.

The governing equations for incompressible turbulent flow are the Reynolds-averaged Navier–Stokes (RANS) equations, closed using the Realizable k−ε turbulence model. This model offers enhanced accuracy in predicting separation and recirculation within intake structures. While the SST k−ω model generally exhibits higher sensitivity to flow curvature in complex wakes, the Realizable k−ε model was adopted for its robust handling of high-velocity shear layers, and its computational stability for prototype-scale simulations [46,47]. The free-surface flow is simulated using the Volume of Fluid (VOF) method, which enables the dynamic tracking of the water-air interface near the gate openings [48,49].

Boundary conditions are specified as follows. The inlet is defined as a pressure inlet with a hydrostatic pressure distribution determined by the upstream water level of each operating condition. The outlet is defined as a mass-flow outlet corresponding to the prototype discharge of 296.7 m^3^/s. The lateral boundaries of the reservoir water body are assigned symmetry conditions (zero normal velocity and flow-variable gradients) to represent an unbounded flow environment and eliminate artificial side-wall effects. All solid surfaces are defined as no-slip walls to accurately capture near-wall velocity gradients and viscous effects. The gravitational acceleration is set to 9.81 m/s^2^ throughout the domain. The flow is assumed to be isothermal at 298.5 K, with the fluid properties are held constant at this temperature. The governing equations are discretized through the application of the finite-volume method. Pressure-velocity coupling is accomplished using the Coupled algorithm. The Least Squares Cell-Based method is employed for gradient calculation. Pressure is discretized using a Modified Body Force Weighted scheme, while momentum and turbulence variables are discretized using Second Order Upwind and First Order Upwind schemes, respectively. The VOF equation is solved using the Compressive interface-capturing scheme to preserve a sharp free surface. To balance computational efficiency with numerical accuracy in this large-scale prototype simulation, scalable wall functions were employed for near-wall treatment. A pseudo-transient iteration with global time-step control is used to enhance convergence stability. Numerical convergence was ensured by requiring scaled residuals to drop below 1 × 10^−4^ while monitoring the overall head loss and reservoir inlet mass flow rate. Simulations were considered converged when these monitored variables exhibited a variation of less than 0.1% over 200 iterations. The maximum iteration limit was set at 50,000.

To assess the mesh independence, Condition 3 is selected as a representative operating condition for grid-convergence analysis. Five mesh configurations are generated with total cell counts of 2.02, 3.20, 5.20, 10.50, and 18.80 million, achieved through proportional refinement in the vicinity of gates and boundary walls. The overall head loss between the intake entrance section and downstream pipe section is used as the evaluation criterion, defined as follows:

(1) $$\Delta H=(\frac{p_{1}}{\rho g}+\frac{{v_{1}}^{2}}{2g}+z_{1})-(\frac{p_{2}}{\rho g}+\frac{{v_{2}}^{2}}{2g}+z_{2})$$

where $p_{1}$ and $p_{2}$ denote the static pressures at the intake and pipe sections, pa; $v_{1}$ and $v_{2}$ are corresponding mean flow velocities, m/s; $z_{1}$ and $z_{2}$ represent the elevations of these sections, m; ρ is the water density, kg/m^3^; and g is the gravitational acceleration, m/s^2^.

The relative deviation of total head loss for each grid density is computed with respect to the finest grid as:

(2) $$\delta_{i}=\frac{\left|\Delta H_{i}-\Delta H_{fine}\right|}{\Delta H_{fine}}\times 100\%$$

where $\delta_{i}$ represents the relative deviation of total head loss, %; $\Delta H_{i}$ is the total head loss obtained with i mesh, and $\Delta H_{fine}$ corresponds to the result from the 18.8 million-cell grid, m.

**Figure 12:**
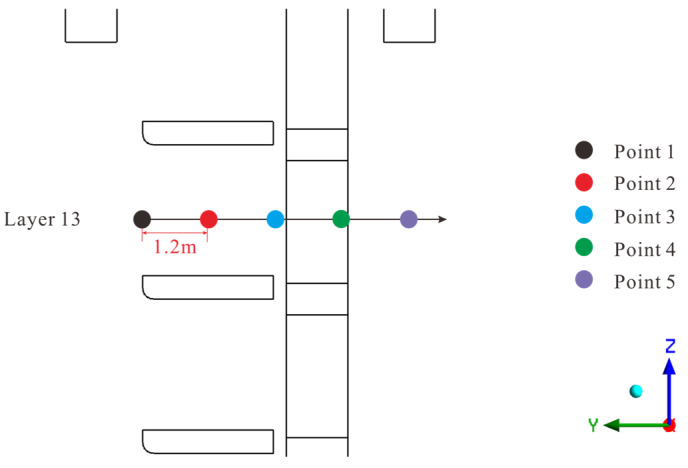
Locations of the velocity monitoring points (P_1_—P_5_) along the centerline.

Given the sensitivity of localized energy dissipation to velocity gradients, a dual-metric approach was employed for the grid independence study. In addition to the global total head loss, the local velocities at five characteristic points were monitored. These points (P_1_—P_5_) were positioned along the centerline of the gate frame in channel_2 (Layer 13) with a spacing of 1.2 m, covering the flow transition from the leading edge to the wake (Figure 12). To verify the near-wall resolution, the average y^+^ value was monitored and maintained at 126.1. This ensures the mesh resides within the logarithmic region, enabling the scalable wall functions to accurately resolve energy dissipation near the boundaries.

**Figure 13:**
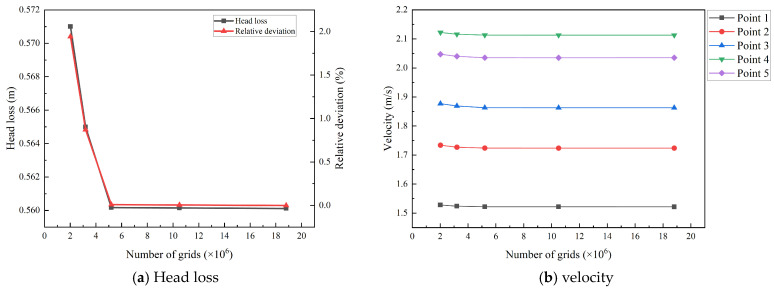
Grid-independence analysis for Condition 3.

As shown in Figure 13a, the total head loss converges gradually with increasing grid density. Simultaneously, the velocity profiles at the five monitoring points in Figure 13b exhibit consistent stabilization. When the total grid number reaches 5.2 million, the relative deviations for both the head loss and all monitored velocities compared to the densest mesh (18.8 million) are less than 0.02%, indicating that the numerical results have achieved mesh independence. Considering both numerical accuracy and computational efficiency, the mesh containing 5.2 million cells was selected for all subsequent simulations.

### 2.3. Entropy Production Theory

Entropy production theory provides a thermodynamic framework for evaluating irreversible energy dissipation in complex hydraulic flows. It links macroscopic hydraulic losses to underlying viscous, turbulent, wall-shear mechanisms, providing a unified basis for assessing flow efficiency and structural performance. According to the second law of thermodynamics, the local entropy transport equation can be written as [29]:

(3) $$\rho (\frac{\partial S}{\partial t}+u_{j}\frac{\partial S}{\partial x_{j}})=\nabla \cdot (\frac{\vec{q}}{T})+\frac{\Phi }{T}+\frac{\Phi_{\Theta }}{T^{2}}$$

where ρ is the fluid density, S is the specific entropy, $\vec{q}$ is the heat flux vector, T is the temperature. The terms Φ and $\Phi_{\Theta }$ denote mechanical and thermal dissipation rate, respectively.

Since the stepless intake targets a specific thermal layer, the internal flow is nearly isothermal. This condition is represented in the numerical model by a constant temperature boundary (298.5 K), which results in a negligible local temperature gradient ∇T. Consequently, the thermal dissipation term $\frac{\Phi_{\Theta }}{T^{2}}$ is negligible, and entropy generation is thus governed by the mechanical dissipation term $\frac{\Phi }{T}$ which captures the irreversible energy loss due to viscous and turbulent effects. The total entropy production rate is therefore expressed as [30,34,35]:

(4) $${\overset{\cdot }{S}}_{Tot}={\overset{\cdot }{S}}_{\bar{D}}^{\prime \prime \prime }+{\overset{\cdot }{S}}_{D^{\prime }}^{\prime \prime \prime }+{\overset{\cdot }{S}}_{W}^{\prime \prime \prime }$$

where ${\overset{\cdot }{S}}_{Tot}$ denote the total entropy production rate, ${\overset{\cdot }{S}}_{\bar{D}}^{\prime \prime \prime }$, ${\overset{\cdot }{S}}_{D^{\prime }}^{\prime \prime \prime }$, and ${\overset{\cdot }{S}}_{W}^{\prime \prime \prime }$ correspond to the entropy production rate caused by direct dissipation (EPDD), turbulent dissipation (EPTD), and wall shear stress (EPWS), respectively.

The entropy production rate induced by direct dissipation (EPDD) can be formulated as Equation (5), which accounts for the irreversible energy loss caused by viscous shear within the mean flow.

(5) $${\overset{\cdot }{S}}_{\bar{D}}^{\prime \prime \prime }=\frac{2\mu }{T}\left[{\left(\frac{\partial \bar{u}}{\partial x}\right)}^{2}+{\left(\frac{\partial \bar{v}}{\partial y}\right)}^{2}+{\left(\frac{\partial \bar{w}}{\partial z}\right)}^{2}\right]+\frac{\mu }{T}\left[{\left(\frac{\partial \bar{v}}{\partial x}+\frac{\partial \bar{u}}{\partial y}\right)}^{2}+{\left(\frac{\partial \bar{w}}{\partial x}+\frac{\partial \bar{u}}{\partial z}\right)}^{2}+{\left(\frac{\partial \bar{v}}{\partial z}+\frac{\partial \bar{w}}{\partial y}\right)}^{2}\right]$$

where μ is the dynamic viscosity and $\bar{u}$, $\bar{v}$, $\bar{w}$ are mean velocity components in the x, y, and z directions, respectively.

The entropy production rate induced by turbulent dissipation (EPTD) can be formulated as Equation (6). This term represents the dissipation resulting from turbulent fluctuations, which is typically the dominant source of loss in high-Reynolds-number flows characterized by flow separation and vortices.

(6) $${\overset{\cdot }{S}}_{D^{\prime }}^{\prime \prime \prime }=\frac{2\mu_{eff}}{T}\left[{\left(\frac{\partial \bar{u}}{\partial x}\right)}^{2}+{\left(\frac{\partial \bar{v}}{\partial y}\right)}^{2}+{\left(\frac{\partial \bar{w}}{\partial z}\right)}^{2}\right]+\frac{\mu_{eff}}{T}\left[{\left(\frac{\partial \bar{v}}{\partial x}+\frac{\partial \bar{u}}{\partial y}\right)}^{2}+{\left(\frac{\partial \bar{w}}{\partial x}+\frac{\partial \bar{u}}{\partial z}\right)}^{2}+{\left(\frac{\partial \bar{v}}{\partial z}+\frac{\partial \bar{w}}{\partial y}\right)}^{2}\right]$$

where $\mu_{eff}$ is the effective dynamic viscosity, incorporating both turbulent viscosity $\mu_{t}$ and dynamic viscosity μ. In Reynolds-averaged computations, the fluctuating velocity gradients contained in this term cannot be directly resolved. Within the Realizable k−ε turbulence model, the viscous dissipation of turbulence is characterized by the turbulent kinetic energy dissipation rate ε; thus, the turbulent entropy production rate ${\overset{\cdot }{S}}_{D^{\prime }}^{\prime \prime \prime }$ is approximated as:

(7) $${\overset{\cdot }{S}}_{D^{\prime }}^{\prime \prime \prime }=\frac{\rho \varepsilon }{T}$$

The entropy production rate induced by wall shear stress (EPWS) can be formulated as Equation (8), representing the frictional losses generated within the thin boundary layers the solid surfaces of walls.

(8) $${\overset{\cdot }{S}}_{W}^{\prime \prime \prime }=\frac{\vec{\tau }\cdot \vec{v}}{T}$$

where $\vec{\tau }$ denotes the wall shear stress vector, $\vec{v}$ denotes the near wall velocity.

The total entropy production within the computational domain is obtained by integrating the local volumetric and surface contributions as shown in Equation (9). This integral provides a macroscopic quantification of all irreversible losses within the system.

(9) $$S_{Tot}=\int_{V}{\overset{\cdot }{S}}_{\bar{D}}^{\prime \prime \prime }dV+\int_{V}{\overset{\cdot }{S}}_{D^{\prime }}^{\prime \prime \prime }dV+\int_{A}{\overset{\cdot }{S}}_{W}^{\prime \prime \prime }dA$$

where $S_{Tot}$ denotes the total entropy production, V is volume, and A is area.

These formulations provide the theoretical basis for computing entropy production in the stepless stratified intake and are used in the subsequent quantitative analysis of regional dissipation and loss mechanisms.

## 3. Results and Discussion

### 3.1. Model Validation and Hydraulic Verification

**Figure 14:**
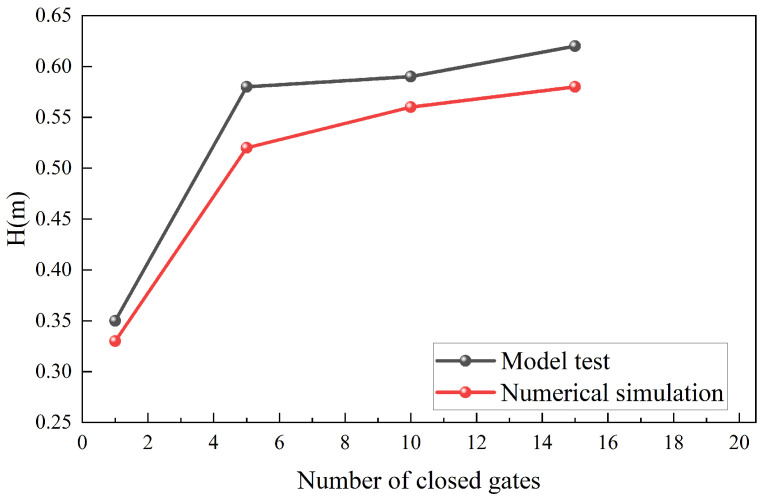
Measured and simulated total head loss for four operating conditions.

**Figure 15:**
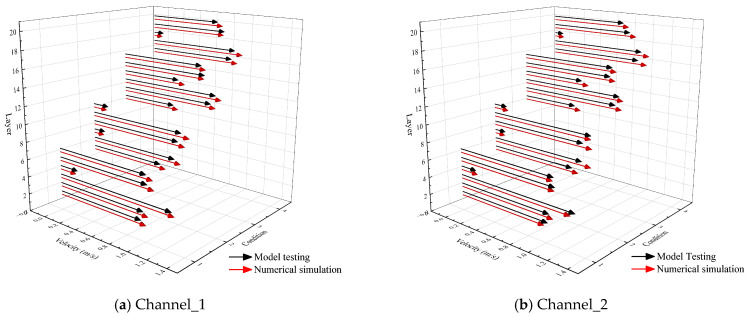
Measured and simulated vertical velocity profiles.

To validate the numerical reliability, a comparative analysis in terms of head loss and flow velocity was carried out between the physical model measurements and the numerical model results, using the experimental configurations described in Section 2.1 and Section 2.2. Figure 14 shows that the simulated head loss reproduces the monotonic increase as the withdrawal layer shifted upward. The maximum discrepancy of 0.06 m occurs under intermediate operating conditions and remains within commonly accepted engineering tolerance. The slightly higher values observed in the physical model are consistent with Reynolds-number distortion in reduced-scale hydraulic experiments, which enhance the relative influence of boundary-layer friction and local losses around gates. By conducting simulations at the prototype scale, the numerical model effectively mitigates these inherent scale effects. This approach allows for the direct resolution of turbulence and boundary layer characteristics at their actual physical dimensions, providing higher fidelity in capturing the localized energy dissipation mechanisms. Figure 15 presents the vertical velocity profiles in Channel 1 and 2. The numerical predictions capture the primary distribution characteristics, including elevated core velocities near the principal intake layers and a gradual decline toward the adjacent layers. For the dominant velocity range (0.5~1.2 m/s), relative deviation is generally within 10%. Localized discrepancies occur at the elevations of the horizontal brace (layers 5, 8, 11, 14, and 18), where geometric blockage promotes shear layer development and small recirculation flow, leading to high-frequency velocity fluctuations in the physical model. For all operating conditions, the simulations accurately reproduce the overall magnitude and vertical trend of the velocity distribution, despite these localized deviations. While the Realizable k−ε model may exhibit a degree of smoothing in the localized turbulent structures within the wakes, the close agreement in global energy loss suggests that the dominant dissipation mechanisms are effectively captured. Beyond the characteristics of the turbulence model, minor discrepancies also stem from inherent uncertainties in the physical model tests. These include the instrumental precision of the flowmeters and water level sensors, environmental fluctuations during data acquisition, and potential scale effects inherent in reduced-scale hydraulic modeling. Given the agreement between the measured and simulated head loss and vertical velocity profiles, the numerical model is considered to provide sufficient predictive fidelity for subsequent entropy-based analysis of irreversible hydraulic dissipation.

### 3.2. Regional Distribution of Entropy Production

**Figure 16:**
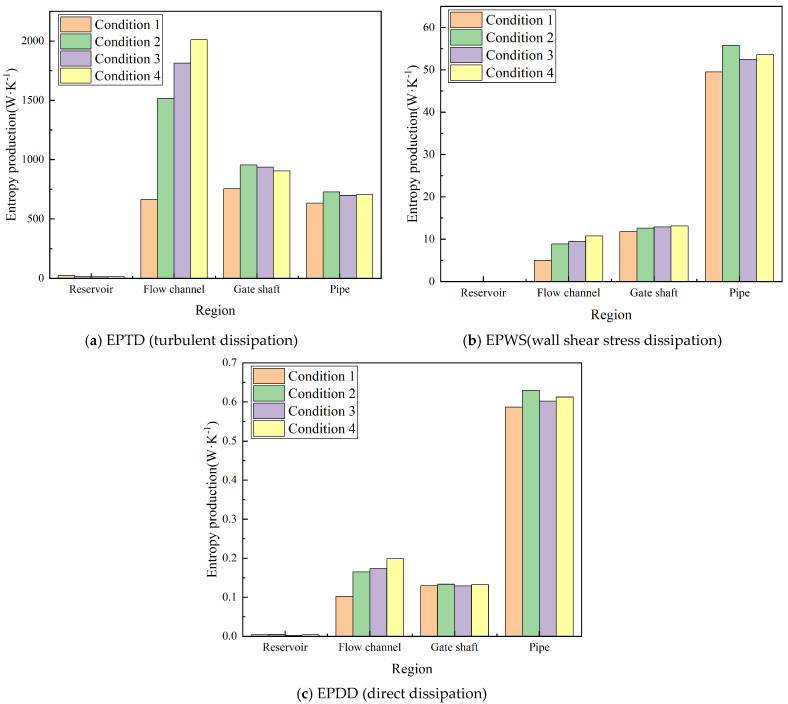
Regional entropy-production components under different operating conditions.

Regional statistics of entropy production are presented in Figure 16. Among the three dissipation components, EPTD overwhelmingly dominates the irreversible losses, accounting for more than 98% of the total under all operating conditions, while EPWS and EPDD are typically two orders of magnitude smaller than EPTD. Spatially, the flow channel and gate shaft constitute the primary sources of entropy production, jointly contributing about 75–89% of the total. The flow channel shows a clear growth trend in EPTD as the withdrawal elevation rises, from approximately 6.6 × 10^2^ W·K^−1^ at Condition 1 to 2.0 × 10^3^ W·K^−1^ at Condition 4, indicating stronger shear-layer interactions and turbulence generation near the intake entrance. The gate shaft exhibits relatively stable dissipation, with values between 7.5 × 10^2^ and 9.5 × 10^2^ W·K^−1^, reflecting its geometric confinement but limited sensitivity to changes in withdrawal elevation. The reservoir contribution is negligible, as its broad cross-section produces low velocity gradients and weak turbulence. The outlet pipe, in contrast, maintains a moderate but nearly invariant EPTD level, between 6.3 × 10^2^ W·K^−1^ and 7.3 × 10^2^ W·K^−1^ across conditions, as the flow becomes progressively aligned with the downstream pipe. While the dominant loss regions are identified at the system scale, a spatially resolved entropy production analysis is still required to uncover the mechanisms that drive energy dissipation.

### 3.3. LEPR-Based Identification of High Entropy Production Regions

**Figure 17:**
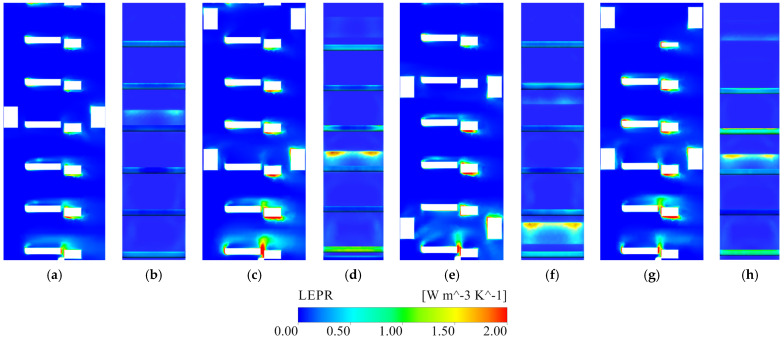
LEPR distribution around the active flap gates in Channel_2. ((**a**,**c**,**e**,**g**) show section A–A (side section) for Conditions 1–4, and (**b**,**d**,**f**,**h**) show section B–B (gate-frame cross-section) for Conditions 1–4, respectively).

**Figure 18:**
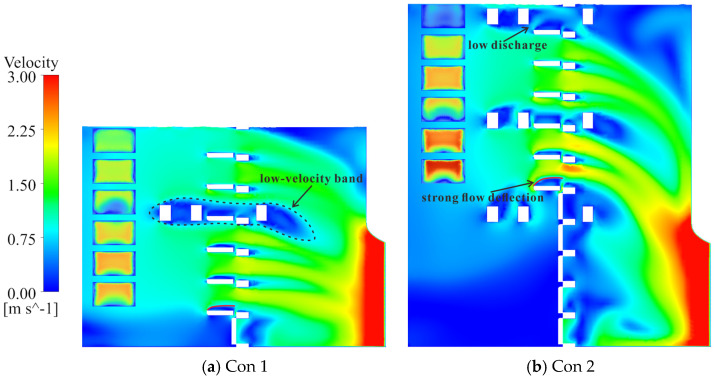

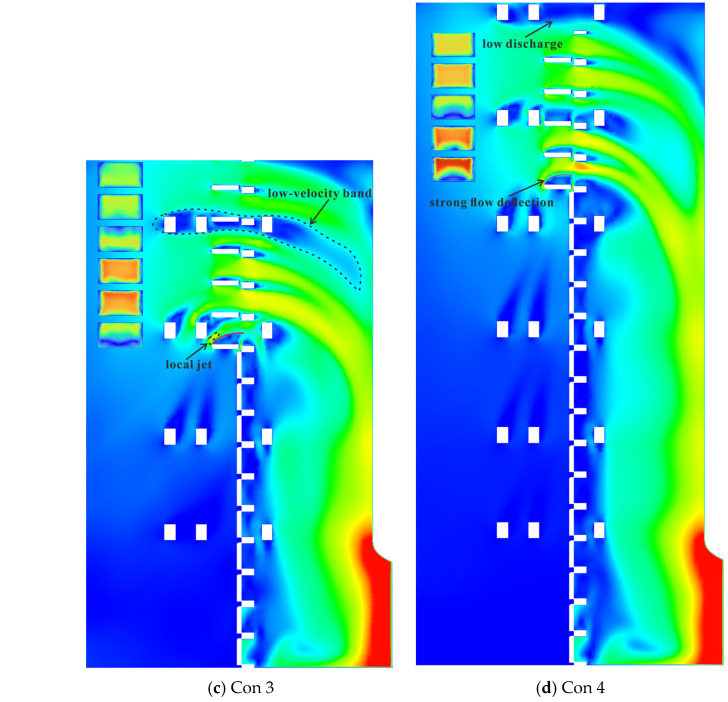
Velocity fields in Channel_2. ((**a**–**d**) show section A–A for Conditions 1–4; in each figure, front-view insets along section B–B at the active gate elevations illustrate the velocity distribution at the corresponding flap-gate openings).

**Figure 19:**
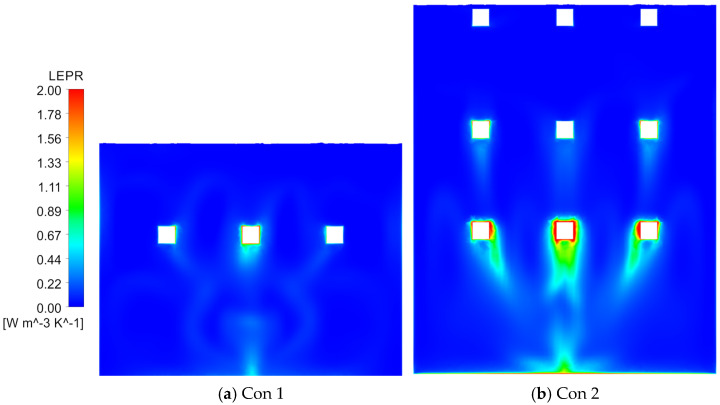

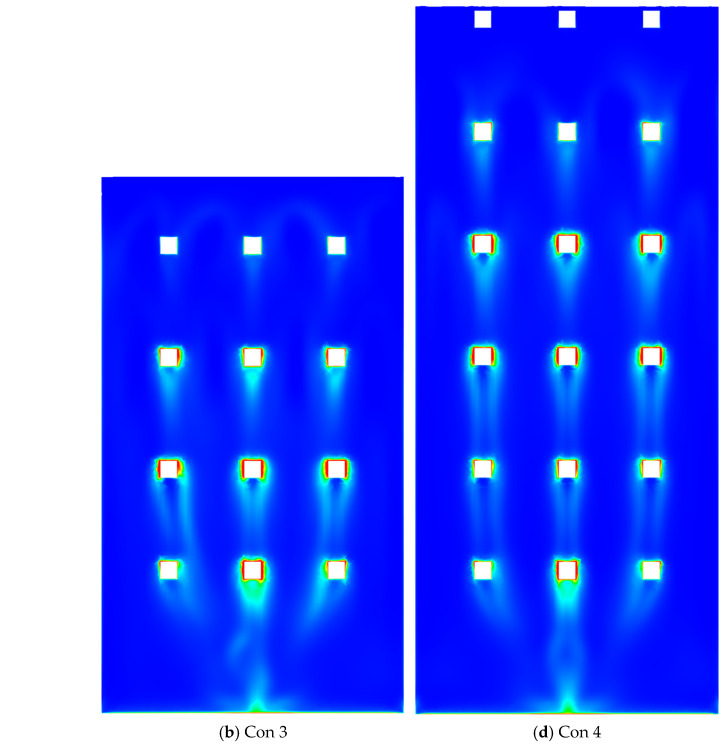
LEPR distribution on section C–C (vertical shaft longitudinal section) under four conditions.

**Figure 20:**
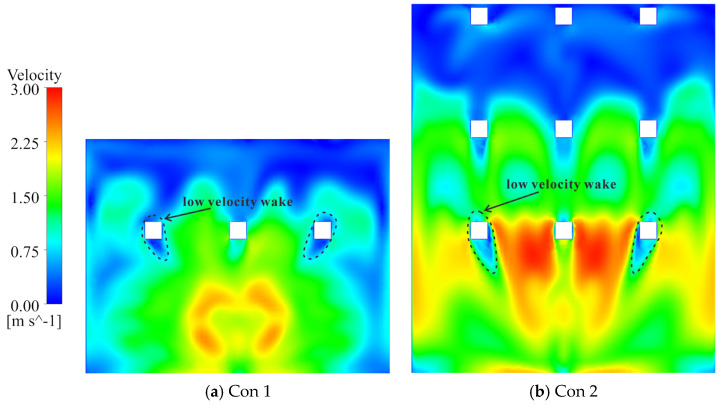

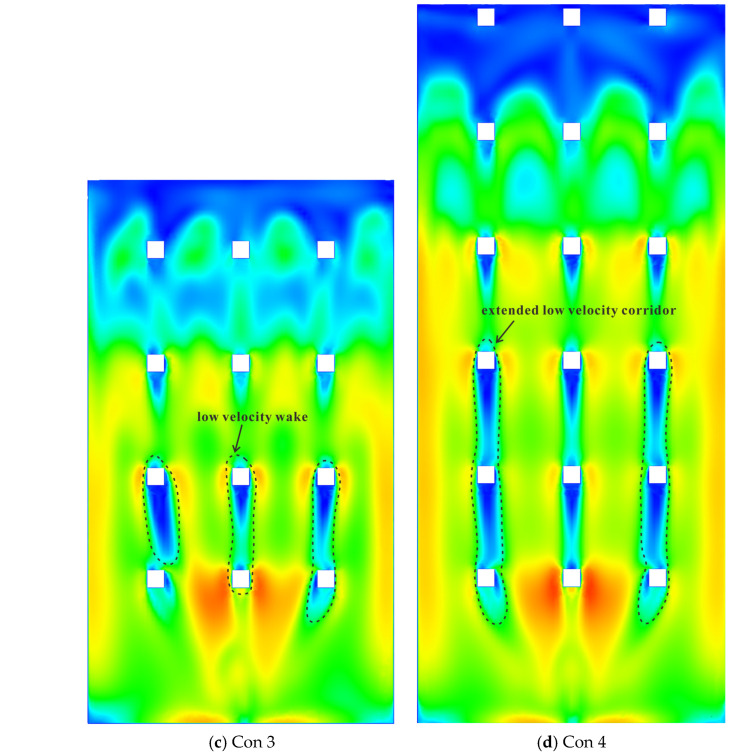
Vertical velocity fields in the vertical shaft on section C–C (vertical shaft longitudinal section) under four conditions.

As shown in Figure 17, Figure 18, Figure 19 and Figure 20, the local entropy-production-rate (LEPR) fields and velocity distributions reveal that irreversible losses in the stepless stratified intake are strongly non-uniform and closely tied to the arrangement of gate leaves and internal braces. The four parallel channels are hydraulically similar, and Channel 2 is therefore selected for detailed illustration as it is representative of the overall behavior under all operating conditions. Under representative operating conditions, high-dissipation zones are primarily concentrated around the lower active gate leaf, the elevations of the horizontal braces (Figure 17) and the vicinity of the vertical braces (Figure 19). These regions exhibit pronounced LEPR peaks aligned with flow-contraction zones and geometric discontinuities, indicating that much of the energy dissipation originates from localized strain amplification rather than from quasi-uniform viscous shear in the core flow.

Within the flow channel, horizontally opened flap gates substantially modify the incoming flow, forcing it to turn around the leaf and forming low-velocity pockets immediately upstream and downstream of the gate (Figure 18). This effect is most evident at the lowest opened layers, where the approach flow impinges almost directly on the bottom gate leaf and produces a strong LEPR concentration on its upstream face. At higher withdrawal elevations, the approach flow is more evenly distributed over depth and the direct stagnation at the leaf front is reduced, leading to weaker local dissipation peaks. Elevated LEPR is consistently observed along the leading edge of the open gate leaf, where the abrupt curvature of the approach streamlines generates intense velocity gradients and local shear layers. These discrete dissipation clusters persist across all withdrawal elevations, demonstrating that entrance curvature at the gate is a robust source of turbulent entropy production.

The horizontal braces introduce an additional, and in many cases more severe, disturbance to the channel flow. At brace elevations (for example, near the fifth-layer gate in Condition 1 and the fourteenth-layer gate in Condition 3), a distinct low-velocity band forms, with large velocity contrasts above and below the gate leaf (Figure 18a,b). The LEPR field highlights secondary peaks attached to the brace surfaces and to the corners of the gate frame, reflecting partial flow separation, velocity-deficit wakes and local recirculation. In Condition 3, where a horizontal brace lies near the center of the opened bottom gate, the brace significantly interferes with the inflow to that gate: a local jet forms in the gap between the gate leaf and the brace, and impinges directly on the upper edge of the frame, producing a strong LEPR hotspot (Figure 17e,f and Figure 18c). The position of the brace also alters discharge partitioning among the gate leaves. The gate located at the brace elevation tends to receive a smaller portion of the total flow, whereas the gates immediately above and below are preferentially supplied, resulting in locally high velocities near the brace layer but a comparatively smoother velocity distribution over the entire cross-section for clustered gate combinations. These features imply not only enhanced local dissipation but also a higher susceptibility of the affected gates to vibration and long-term fatigue under continuous operation.

After the flow passes through the gate openings and enters the vertical shaft, the influence of the vertical braces becomes more prominent. The flow descends under gravity and accelerates, leading to increasing LEPR levels around the lower braces. The LEPR field forms a continuous near-wall band along the confining walls, punctuated by strong peaks at the positions of the vertical braces (Figure 19). Behind each brace, a low-velocity wake and recirculation zone are observed, producing localized high-entropy-production cores (Figure 20). Because the vertical braces are arranged in a single array along the shaft wall, there is little lateral interaction among their wakes. In the vertical direction, however, the low-velocity wakes shed from upstream braces are converging downward and overlap with those generated by the lower braces, forming an extended corridor of reduced velocity and elevated LEPR. In Condition 4, for instance, a nearly continuous low-velocity corridor develops between the first and third brace levels, where the combined effect of multiple wakes creates an extended region of elevated dissipation and reduced flow momentum (Figure 19d and Figure 20d).

Overall, the LEPR distribution indicates that major energy dissipation within the stepless stratified intake arises from two coupled geometric effects: curvature-induced shear amplification at the gate entrance and flow separation and wake formation associated with the horizontal and vertical braces. The horizontal braces primarily reorganize the vertical distribution of discharge and generate low-velocity bands with strong velocity contrasts across the gate leaf, while the vertical braces control the development and superposition of wakes within the vertical shaft. These mechanisms act concurrently to produce discrete yet persistent dissipation hotspots that dominate the hydraulic efficiency of the system and set the geometric targets for the refinement schemes evaluated in Section 3.4.

### 3.4. Entropy-Based Refinement of Hydraulic Performance

**Figure 21:**
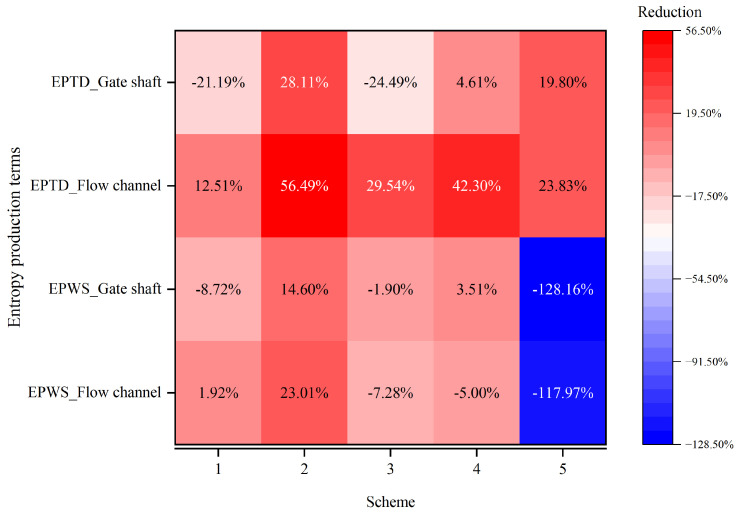
Relative changes in EPTD and EPWS in the flow channel and gate shaft for the original configuration and refinement Schemes 1–5 (Condition 4).

**Figure 22:**
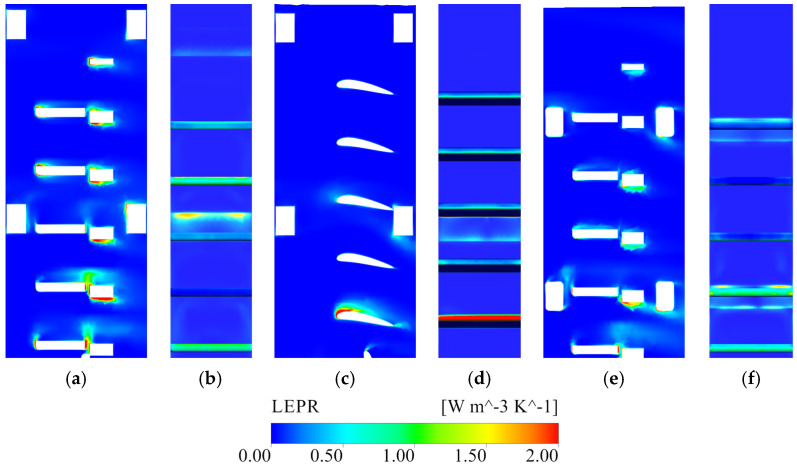
LEPR distribution around the active flap gates in Channel 2 under Condition 4. ((**a**,**c**,**e**) show section A–A for origin, scheme 1, and scheme 5; (**b**,**d**,**f**) show section B–B for origin, scheme 1, and scheme 5).

**Figure 23:**
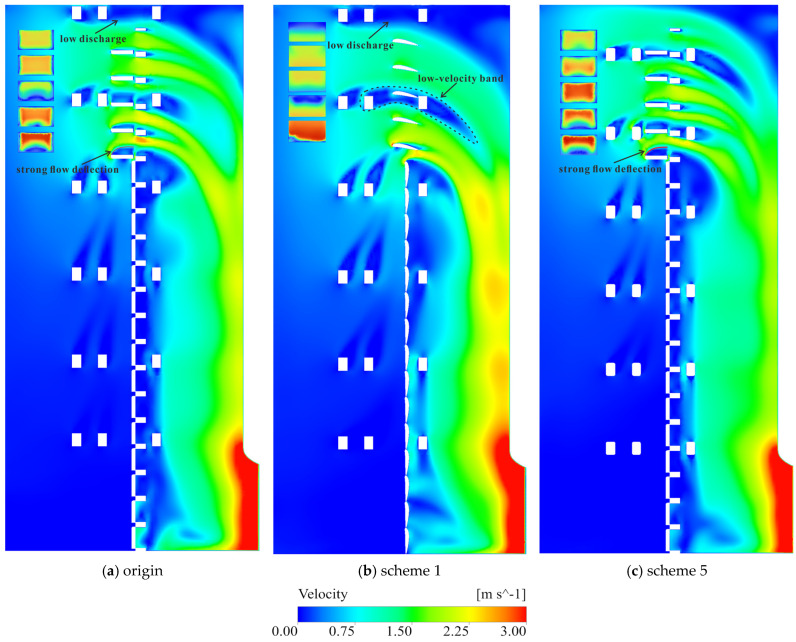
Velocity fields in Channel_2 under condition 4. ((**a**–**c**) show section A–A for origin, scheme 1, and scheme 5; in each figure, front-view insets along section B–B at the active gate elevations illustrate the velocity distribution at the corresponding flap-gate openings).

**Figure 24:**
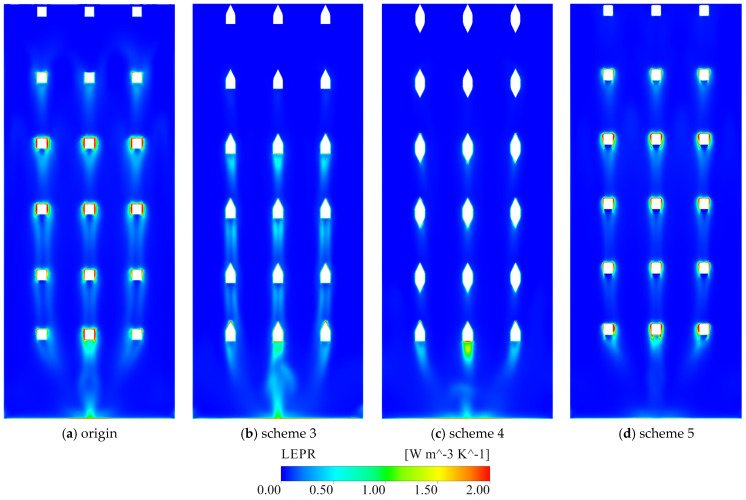
LEPR distribution on section C–C under condition 4.

**Figure 25:**
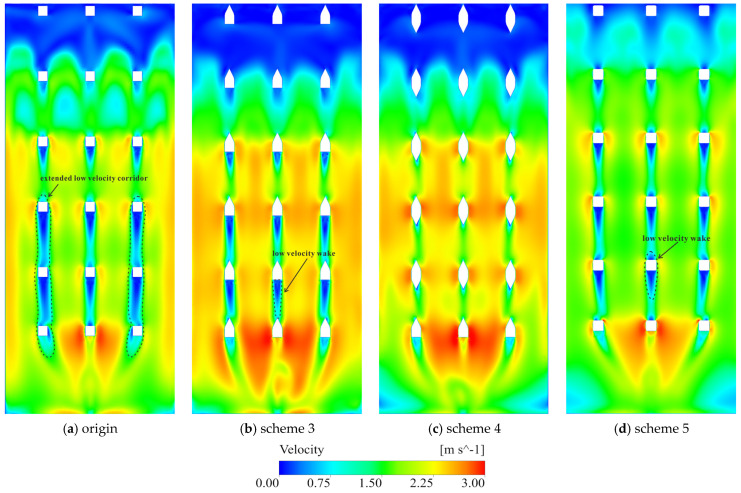
Vertical velocity fields on section C–C under condition 4.

Guided by the LEPR diagnostics in Section 3.3, five geometric modifications were evaluated under Condition 4, the most dissipative configuration, to act directly on the identified mechanisms—moderating shear amplification at the gate entrance and weakening brace-induced wakes, while preserving conveyance capacity. The assessment combines LEPR and velocity fields with regional EPTD and EPWS values to quantify how dissipation is redistributed between the flow channel and the gate shaft (Figure 21, Figure 22, Figure 23, Figure 24 and Figure 25). The tested schemes include: (1) replacement of the flap gate with an NACA-profile leaf; (2) removal of the vertical brace; (3) addition of an isosceles triangular component on the upstream side of the vertical brace; (4) addition of isosceles triangular components on both the upstream and downstream sides of the vertical brace; and (5) alignment of the horizontal and vertical braces with the gate-frame boundary combined with a 150 mm corner rounding. Scheme 2 is structurally infeasible and is treated only as an idealized upper-bound reference in which brace-induced disturbances are fully removed; its LEPR and velocity fields are nearly uniform and add little further insight, so only regional entropy-production statistics are reported.

From the perspective of gate-entrance refinement, scheme 1 and scheme 5 have distinct hydraulic implications. In scheme 1, the NACA-profile gate leaf streamlines the leading edge and alleviates the strong shear amplification previously observed at the entrance. The approach flow turns more smoothly around the leaf, the LEPR contours show a thinner high-value band at the gate entrance and a more continuous transition into the vertical shaft, while the core high-velocity region expands (Figure 23b). Quantitatively, this localized streamlining reduces the EPTD within the flow channel by 12.51%. However, the overall efficiency gain is partially counterbalanced by secondary hydraulic effects. First, the suppression of flow separation, which would otherwise function as a dissipative buffer, leads to a more direct and unattenuated jet impingement on the gate’s leading edge, particularly at the lowest gate (Figure 22c,d). Second, the streamlined geometry allows the approach flow to sustain its momentum and enter the gate shaft with a higher velocity magnitude; this more energetic flow subsequently intensifies localized turbulent dissipation, increasing the EPTD within the shaft region by 21.19% (Figure 21). Furthermore, the low-velocity wake regions induced by the horizontal braces remain largely unchanged at their respective elevations, and the non-uniformity in gate-to-gate discharge distribution persists. In net terms, scheme 1 primarily results in a spatial reallocation of entropy production from the upstream channel to the shaft, illustrating that entrance streamlining alone has a limited impact on overall energy loss without mitigating internal structural interference.

In scheme 5, the combined brace-alignment and edge-rounding design does not completely eliminate the strong shear and local separation at the leaf front (Figure 22e,f), but it significantly mitigates the disturbances generated by the horizontal braces. Aligning the horizontal and vertical braces with the gate frame and rounding their edges improves streamline continuity through the gate–vertical shaft transition and weakens secondary curvature at member intersections. Velocity fields show that the previously continuous low-velocity band across the gate opening is broken up; the flow bends more gently around the rounded brace edges, and the inflow to each flap gate becomes smoother. The discharge distribution among the different gate levels is improved, with no single gate at a brace elevation experiencing a persistent velocity deficit (Figure 23c). In the original design, the blockage effect caused by the horizontal braces obstructed the upper 3–4 m of the water column, limiting the intake’s effectiveness in capturing the warmest surface layers. By adopting the combined brace-alignment (scheme 5), this obstruction is removed, allowing the intake to fully utilize the 18 m design withdrawal range. A comparative analysis of the velocity profiles indicates that scheme 5 achieves a more uniform discharge distribution across the gate leaf, ensuring the intake more accurately and effectively targets the intended thermal layers. Regionally, EPTD decreases in both the flow channel and the gate shaft, while EPWS exhibits a modest increase due to stronger near-wall attachment (Figure 21). Because EPWS remains much smaller than EPTD, the net effect is a favorable reduction in total irreversible loss. Considering both hydraulic performance and structural feasibility, scheme 5 provides a robust and practically attractive refinement for the gate-entrance region and the associated horizontal-brace disturbances.

The schemes targeting the vertical braces (schemes 2–4, together with the vertical-brace rounding in scheme 5) focus on suppressing wake development within the shaft. In the original configuration, the vertical braces generate strong LEPR peaks on their upstream faces and extended low-velocity wakes and recirculation zones downstream (Figure 25a). Because the braces are arranged in a single array along the shaft wall, their wakes exhibit little lateral interaction. In the vertical direction, however, the low-velocity wakes shed from the upper braces are converging downward and overlap with those generated by the lower braces, forming a nearly continuous low-velocity corridor with elevated entropy production in the lower part of the shaft (Figure 24a).

Removing the vertical brace (scheme 2) almost completely eliminates the high-LEPR cores previously attached to it and substantially simplifies the shaft flow. The LEPR field becomes more uniform, and EPTD decreases in both the flow channel and the shaft, yielding a markedly lower total entropy production compared with the original scheme. This confirms that the vertical brace is a primary driver of dissipation through its interference with the main downward jet and the associated shear amplification (Figure 21).

In scheme 3, an isosceles triangular component is attached only to the upstream side of the vertical brace. This upstream fairing alleviates direct stagnation at the brace head and allows the approaching flow to attach and accelerate more easily along the brace surface (Figure 25b). EPTD in the flow channel is consequently reduced, and LEPR peaks on the upstream face of the brace are weakened (Figure 24b). However, the trailing edge of the brace remains bluff, and separation still occurs there. A distinct tail vortex develops downstream of the brace, promoting the growth of a low-velocity recirculation zone and increasing EPTD in the gate shaft. The total entropy production is reduced relative to the original case, but the mitigation is partial and the shaft dissipation remains significant (Figure 21).

Scheme 4 adds triangular components on both the upstream and downstream sides of the vertical brace. This dual-sided modification further suppresses wake formation at the brace tail: the flow remains attached over most of the brace surface, and the LEPR distribution around the brace becomes much more uniform. High-entropy-production cores near the brace are strongly diminished, and the downstream wake shrinks (Figure 24c). EPTD in the flow channel is substantially reduced, and gate shaft EPTD also decreases slightly (Figure 21). At the same time, the focusing of momentum into the shaft core leads to higher peak velocities, as the discharge is more tightly confined to a coherent jet and lateral diffusion is suppressed. This local acceleration is beneficial for dissipation mitigation but may increase local structural loading on the shaft walls and downstream components (Figure 25c).

The rounding applied in scheme 5 also acts on the vertical braces. Rounding the brace edges reduces the sharp separation at the brace tips and encourages the lateral side streams to converge toward the brace, which reduces the size and intensity of the low-velocity wake compared with the original scheme (Figure 24d and Figure 25d). In contrast to schemes 3 and 4, the acceleration of the shaft core flow is more moderate, so that EPTD is reduced without an excessive increase in peak velocity (Figure 21). In combination with the improvements around the horizontal braces and gate entrance, scheme 5 achieves a relatively balanced dissipation field, with weakened local hotspots and improved discharge sharing, while remaining structurally realistic.

Comparison of entropy production across all five refinement schemes demonstrates that geometric modification primarily redistributes, rather than uniformly suppresses, energy dissipation. High-dissipation zones near the gate entrance and internal braces become weaker and more spatially dispersed, resulting in a smoother energy-transfer path through the intake. This redistribution, rather than absolute attenuation, controls the overall hydraulic performance, linking efficiency directly to the spatial pattern of irreversible losses. Among the tested configurations, the strength of flow–structure interaction emerges as the governing factor. Reducing the penetration of braces into the main flow and softening sharp geometric discontinuities—through streamlining, dual-sided fairings or alignment-and-rounding designs—limits strain accumulation in the flow channel and avoids the formation of additional dissipation zones in the gate shaft. In practice, layouts that preserve a coherent core flow and weaken strong flow–structure interactions perform better than configurations optimized solely with respect to overall head-loss reduction, and they offer a more comprehensive basis for refining stepless stratified intake structures. Furthermore, since the discharge is constant across all conditions and the variable is limited to the intake layer, the performance gains of scheme 5 are intrinsically tied to the structural geometry of the braces and gate frames. Because these geometric features trigger dissipation regardless of the intake elevation, their refinement ensures that scheme 5 remains the most effective configuration across the entire operating spectrum, providing a robust solution for all intake layers.

From a practical engineering standpoint, the five schemes present different trade-offs. Scheme 2 (removal of the vertical brace), while hydraulically efficient, may significantly compromise the structural strength and load-bearing capacity of the gate frame, making it less viable for high-head projects. Scheme 1 (NACA-profile leaf) and scheme 5 (corner rounding and alignment) involve complex curved surfaces that increase construction difficulty and initial fabrication costs compared to the simpler triangular additions in schemes 3 and 4. However, the 150 mm corner rounding in scheme 5 is well within the capabilities of modern concrete casting or steel fabrication. Ultimately, the streamlined nature of scheme 5 is superior in suppressing flow-induced vibrations and cavitation risk, thereby reducing long-term maintenance costs. Consequently, scheme 5 is recommended as the optimal balance between hydraulic excellence and structural longevity. Furthermore, it should be emphasized that these geometric refinements focus on localized flow streamlining and do not alter the vertical position or opening height of the intake. Therefore, the selective withdrawal characteristics and the resulting water temperature regulation capability remain unchanged. This ensures that the structural efficiency gains do not compromise the broader ecological objective of providing optimal thermal conditions for downstream aquatic ecosystems.

## 4. Conclusions

This study investigated the internal energy-loss mechanisms of a stepless stratified intake using physical-model tests and a validated three-dimensional numerical model combined with entropy-production theory. The main conclusions are as follows:

(1) At the system scale, turbulent entropy production dominates the loss budget, typically exceeding 98% of the total, while EPWS and EPDD are two orders of magnitude smaller. Entropy production is concentrated in the flow channel and gate shaft (about 75–89% of the total), with negligible contribution from the reservoir and nearly invariant dissipation in the outlet pipe, indicating that turbulence-generating structures in the approach channel and gate shaft control the overall hydraulic performance of the stepless stratified intake.
(2) LEPR fields show that major irreversible losses arise from curvature-induced shear amplification at the gate entrance and from separation and wake formation around horizontal and vertical braces. Horizontally opened flap gates create high-LEPR clusters along the leading edge and adjacent low-velocity pockets, while horizontal braces generate low-velocity bands and strong velocity contrasts across gate leaves, and vertically aligned braces produce overlapping wakes that form an extended low-velocity corridor in the shaft.
(3) Entropy-based comparison of five geometric schemes indicates that refinement primarily redistributes, rather than uniformly suppresses, energy dissipation. NACA profiling of the gate leaf mainly shifts dissipation from the approach channel to the shaft and yields only a modest net reduction, whereas brace fairings and alignment-and-rounding designs weaken stagnation and wakes; among the practically feasible configurations, the combined brace-alignment and edge-rounding scheme provides the most balanced improvement, mitigating low-velocity bands, improving discharge sharing among gate levels, and markedly reducing total entropy production without excessive local acceleration.
(4) From a design standpoint, the performance of stepless stratified intakes is governed by the spatial organization of entropy production rather than by bulk head-loss values alone. Layouts that maintain a coherent core jet, weaken strong flow–structure interactions at gate leaves and braces, and avoid long low-velocity corridors outperform configurations optimized only for global loss coefficients, and the entropy-based analysis framework adopted here offers a thermodynamically consistent basis for refining entrance geometry and brace arrangements in future designs.

The present findings highlight the value of entropy-production diagnostics for the hydraulic design of deep-reservoir intakes: by linking efficiency to the spatial pattern of irreversible losses, the method allows critical flow–structure interactions to be targeted directly. Future work may extend this framework to multi-unit layouts, unsteady operating scenarios, and coupled hydro-thermal–ecological refinement and may further incorporate structural response to assess vibration and fatigue risks associated with long-term operation of stepless stratified intake structures.

## Figures and Tables

**Table 1:** Overall model size of water inlet.

	Length (m)	Width (m)	Height (m)	Gate Height (m)	Gate Width (m)	Re (×10^5^)
Prototype	200	125	80	6	4.5	201.96
Model	10	6.25	4	0.3	0.225	2.258

**Table 2:** Prototype inlet calculation condition.

Condition ID	Closed Gates per Channel	Total Closed Gates (4 Channels)	Flow Range	Water Depth (m)
1	1	4	Layer 2~7	21
2	5	20	Layer 6~11	33
3	10	40	Layer 11~16	48
4	15	60	Layer 15~20 + 3 m above lay 20	63

(Each channel contains 20 flap gates, numbered from 1 (bottom) to 20 (top). “Closed gates per channel” refers to the number of flap gates kept closed from the lowest gate upward in each channel).

## Data Availability

The data are available from the authors upon reasonable request.

## References

[1] Kim S.K., Choi S.-U. (2021). Assessment of the impact of selective withdrawal on downstream fish habitats using a coupled hydrodynamic and habitat modeling. J. Hydrol..

[2] Wang X., Wang P., Deng Y., Xing X., Yuan Q., Du C., Gan J., Zheng Y., Liu Y., Xia Y. (2024). Impacts of cascade hydropower development on river ecosystem homeostasis: A review. J. Hydrol..

[3] He F., Zarfl C., Tockner K., Olden J.D., Campos Z., Muniz F., Svenning J.-C., Jähnig S.C. (2024). Hydropower impacts on riverine biodiversity. Nat. Rev. Earth Environ..

[4] Rheinheimer D.E., Null S.E., Lund J.R. (2015). Optimizing selective withdrawal from reservoirs to manage downstream temperatures with climate warming. J. Water Resour. Plan. Manag..

[5] Alizadeh F., Niksokhan M.H., Nikoo M.R., Mishra A., Al-Wardy M., Al-Rawas G. (2025). Enhancing water security through integrated decision-making and selective withdrawal for sustainable reservoir management. Sci. Rep..

[6] Saadatpour M., Javaheri S., Afshar A., Sandoval Solis S. (2021). Optimization of selective withdrawal systems in hydropower reservoir considering water quality and quantity aspects. Expert Syst. Appl..

[7] Wang H., Deng Y., Yang Y., Lu J., Tuo Y., Yan Z., Chen M. (2024). Optimization of selective withdrawal strategy in a warm monomictic reservoir based on thermal stratification. Ecol. Indic..

[8] Hu G., Yang Z., Yue Y., Bai F., Ren Y. (2025). Joint thermal regulation by selective withdrawal in serial cascade reservoir systems effectively improves reservoir and downstream ecological health. Water Res..

[9] Castelletti A., Yajima H., Giuliani M., Soncini-Sessa R., Weber E. (2014). Planning the optimal operation of a multioutlet water reservoir with water quality and quantity targets. J. Water Resour. Plan. Manag..

[10] Deng Y., Tuo Y., Li J., Li K., Li R. (2011). Spatial-temporal effects of temperature control device of stoplog intake for Jinping I hydropower station. Sci. China Technol. Sci..

[11] Gray R., Jones H.A., Hitchcock J.N., Hardwick L., Pepper D., Lugg A., Seymour J.R., Mitrovic S.M. (2019). Mitigation of cold-water thermal pollution downstream of a large dam with the use of a novel thermal curtain. River Res. Appl..

[12] Duka M.A., Shintani T., Yokoyama K. (2021). Thermal stratification responses of a monomictic reservoir under different seasons and operation schemes. Sci. Total Environ..

[13] Wang H., Deng Y., Yan Z., Yang Y., Tuo Y. (2023). Thermal response of a deep monomictic reservoir to selective withdrawal of the upstream reservoir. Ecol. Eng..

[14] Lu Y., Tuo Y., Xia H., Zhang L., Chen M., Li J. (2023). Prediction model of the outflow temperature from stratified reservoir regulated by stratified water intake facility based on machine learning algorithm. Ecol. Indic..

[15] Hu G., Yang Z., Lu J., Bai F. (2025). Thermal response of deep monomictic reservoir under different selective withdrawal types. J. Environ. Manag..

[16] Mi C., Sadeghian A., Lindenschmidt K.-E., Rinke K. (2019). Variable withdrawal elevations as a management tool to counter the effects of climate warming in Germany’s largest drinking water reservoir. Environ. Sci. Eur..

[17] Soyer E., Bayram H., Canıgeniş N., Eren O. (2023). Decision support system for selective withdrawal in water supply reservoirs: An approach based on thermal stratification. Water Qual. Res. J..

[18] Wang H., Deng Y., Yang Y., Chen M., Wang X., Tuo Y. (2024). Future projections of thermal regimes and mixing characteristics in a monomictic reservoir under climate change. Sci. Total Environ..

[19] Mi C., Tilahun A.B., Flörke M., Dürr H.H., Rinke K. (2024). Climate warming effects in stratified reservoirs: Thorough assessment for opportunities and limits of machine learning techniques versus process-based models in thermal structure projections. J. Clean. Prod..

[20] Wang X., Wang W., He Y., Zhang S., Huang W., Woolway R.I., Shi K., Yang X. (2023). Numerical simulation of thermal stratification in lake qiandaohu using an improved WRF-lake model. J. Hydrol..

[21] Mi C., Rinke K., Shatwell T. (2024). Optimizing selective withdrawal strategies to mitigate hypoxia under water-level reduction in Germany’s largest drinking water reservoir. J. Environ. Sci..

[22] He W., Wang H., Zhang J., Xu H., Xiao Y. (2023). Diurnal variation characteristics of thermal structure in a deep reservoir and the effects of selective withdrawal. J. Environ. Manag..

[23] Liu L., Tuo Y., Xia H., Deng Y., Zhang X., Wang H. (2023). Assessment of stoplog gates’ operational effectiveness for improving discharged-water temperatures during the thermal stratification period in a reservoir. Water.

[24] Yang Z., Wei C., Liu D., Lin Q., Huang Y., Wang C., Ji D., Ma J., Yang H. (2022). The influence of hydraulic characteristics on algal bloom in three gorges reservoir, China: A combination of cultural experiments and field monitoring. Water Res..

[25] Song Y., Chen M., Li J., Zhang L., Deng Y., Chen J. (2023). Can selective withdrawal control algal blooms in reservoirs? The underlying hydrodynamic mechanism. J. Clean. Prod..

[26] Zheng T., Sun S., Liu H., Xia Q., Zong Q. (2016). Optimal control of reservoir release temperature through selective withdrawal intake at hydropower dam. Water Supply.

[27] Yang S., Zhang Z., Ji Q., Liang R., Li K. (2023). Study on the water temperature distribution characteristics of a mixed pumped storage power station reservoir: A case study of Jinshuitan reservoir. Renew. Energy.

[28] Wang Y., Zhong X., Wang B., Hu J., Bao Z., Xu G., Liu Y., Han X. (2020). Numerical computation of multi-level intake of diversion project. IOP Conf. Ser. Earth Environ. Sci.

[29] Kock F., Herwig H. (2004). Local entropy production in turbulent shear flows: A high-Reynolds number model with wall functions. Int. J. Heat Mass Transf..

[30] Herwig H., Kock F. (2006). Direct and indirect methods of calculating entropy generation rates in turbulent convective heat transfer problems. Heat Mass Transf..

[31] Balaji C., Hölling M., Herwig H. (2007). Entropy generation minimization in turbulent mixed convection flows. Int. Commun. Heat Mass Transf..

[32] Zhou L., Hang J., Bai L., Krzemianowski Z., El-Emam M.A., Yasser E., Agarwal R. (2022). Application of entropy production theory for energy losses and other investigation in pumps and turbines: A review. Appl. Energy.

[33] Gong R., Wang H., Chen L., Li D., Zhang H., Wei X. (2013). Application of entropy production theory to hydro-turbine hydraulic analysis. Sci. China Technol. Sci..

[34] Yu A., Tang Q., Chen H., Zhou D. (2021). Investigations of the thermodynamic entropy evaluation in a hydraulic turbine under various operating conditions. Renew. Energy.

[35] Li D., Wang H., Qin Y., Han L., Wei X., Qin D. (2017). Entropy production analysis of hysteresis characteristic of a pump-turbine model. Energy Convers. Manag..

[36] Yan X., Kan K., Zheng Y., Chen H., Binama M. (2022). Entropy production evaluation within a prototype pump-turbine operated in pump mode for a wide range of flow conditions. Processes.

[37] Shi F., Zhang D., Wang P., Wang X., Feng C. (2025). Analysis of the Energy Loss Mechanism in Hydraulic Turbines with Different Guide-Vane Numbers Based on Entropy Generation Theory. Processes.

[38] Hou H., Zhang Y., Li Z., Jiang T., Zhang J., Xu C. (2016). Numerical analysis of entropy production on a LNG cryogenic submerged pump. J. Nat. Gas Sci. Eng..

[39] Ji L., Li W., Shi W., Tian F., Agarwal R. (2020). Diagnosis of internal energy characteristics of mixed-flow pump within stall region based on entropy production analysis model. Int. Commun. Heat Mass Transf..

[40] Pei J., Meng F., Li Y., Yuan S., Chen J. (2016). Effects of distance between impeller and guide vane on losses in a low head pump by entropy production analysis. Adv. Mech. Eng..

[41] Zhang F., Appiah D., Hong F., Zhang J., Yuan S., Adu-Poku K.A., Wei X. (2020). Energy loss evaluation in a side channel pump under different wrapping angles using entropy production method. Int. Commun. Heat Mass Transf..

[42] Wang X., Yan Y., Wang W.-Q., Hu Z.-P. (2023). Evaluating energy loss with the entropy production theory: A case study of a micro horizontal axis river ducted turbine. Energy Convers. Manag..

[43] Lin T., Li X., Zhu Z., Xie J., Li Y., Yang H. (2021). Application of enstrophy dissipation to analyze energy loss in a centrifugal pump as turbine. Renew. Energy.

[44] Wan B., Zhang Z., Wang Y., Xie Q., Zhang X., Si Q. (2025). Energy distribution in radial-inflow turbines following enthalpy gradient magnitude method. Appl. Therm. Eng..

[45] Wu F., Zhang F., Chen K., Xu X., Yuan S. (2025). Investigation of the impact of trapezoidal suction surface impeller on internal flow and energy conversion in side channel pumps. Energy.

[46] Rezaeiha A., Montazeri H., Blocken B. (2019). On the accuracy of turbulence models for CFD simulations of vertical axis wind turbines. Energy.

[47] Liu M., Jiang C., Khoo B.C., Zhu H., Gao G. (2024). A cell-based smoothed finite element model for the analysis of turbulent flow using realizable k-ε model and mixed meshes. J. Comput. Phys..

[48] Hirt C.W., Nichols B.D. (1981). Volume of fluid (VOF) method for the dynamics of free boundaries. J. Comput. Phys..

[49] Zhang X., Tang F., Pavesi G., Hu C., Song X. (2024). Influence of gate cutoff effect on flow mode conversion and energy dissipation during power-off of prototype tubular pump system. Energy.

